# How do cardiovascular risk factors correlate with post-stroke cognitive function: Directly or indirectly through stroke severity?

**DOI:** 10.3389/fneur.2022.917295

**Published:** 2022-08-05

**Authors:** Jianian Hua, Yixiu Zhou, Licong Chen, Xiang Tang, Shanshan Diao, Qi Fang

**Affiliations:** ^1^Department of Neurology, The First Affiliated Hospital of Soochow University, Suzhou, China; ^2^Department of Emergency, Children's Hospital of Soochow University, Suzhou, China

**Keywords:** stroke, cognitive function, cross-sectional study, moderated and mediation analysis, risk factors

## Abstract

**Objectives:**

Cognitive impairment may affect one-third of stroke survivors. Cardiovascular risk factors and stroke severity were known to be associated with cognitive function after stroke. However, it is unclear whether cardiovascular risk factors directly affect cognition after stroke, indirectly affect cognition by changing stroke severity, or both. Moreover, the effect of a combination of hypertension and diabetes mellitus was conflicting. We aimed to investigate the multiple direct and indirect associations and inspire potential intervention strategies.

**Materials and methods:**

From February 2020 to January 2021, 350 individuals received cognitive tests within 7 days after incident stroke. Cognitive tests were performed using the Chinese version of the Mini-Mental State Examination (MMSE). A moderated mediation model was constructed to test the indirect associations between cardiovascular and demographic risk factors and cognition mediated through stroke severity, the direct associations between risk factors and cognition, and the moderating effects of hypertension and diabetes.

**Results:**

Age (estimate, −0.112), atrial fibrillation (estimate, −4.092), and stroke severity (estimate, −1.994) were directly associated with lower cognitive function after stroke. Vascular disease (estimate, 1.951) and male sex (estimate, 2.502) were directly associated with better cognition after stroke. Higher education level was associated with better cognition directly (estimate, 1.341) and indirectly (estimate, 0.227) through stroke severity. The combination of hypertension decreased the magnitude of the negative association between atrial fibrillation and cognition (estimate, from −4.092 to −3.580).

**Conclusion:**

This is the first Chinese study exploring the moderated and mediating associations between cardiovascular risk factors, stroke severity, and cognitive function after stroke. Age, female sex, and atrial fibrillation were directly associated with lower cognition after stroke. The combination of hypertension might have a positive effect on cognition.

## Introduction

Currently, the incidence rate of ischemic stroke is growing (1). It is well known that stroke is associated with acute cognitive decline and an increased risk of dementia (2, 3). Among those who suffer from a mild stroke, even though their motor functions might recover through rehabilitation training, their cognitive decline is hard to reverse and has been frequently overlooked. Lower cognition is associated with poor health and quality of life, impairments in functional abilities, and increased medical costs (4). For most, losing one's cognitive abilities is feared more than physical ability. Decreased cognition inflicts a great burden on caregivers and global health (5, 6). Therefore, learning modifiable risk factors and exploring novel complex associations are important steps for developing strategies aimed at maintaining healthy cognitive aging among stroke survivors.

One question is why do some stroke survivors have worse cognitive trends than others? The acute cognitive decline after stroke is obviously associated with acute brain damage (i.e., lesions) and subsequent response (i.e., the inflammatory factors) (7). Meanwhile, recent studies reported that compared with participants without stroke, those who experienced incident stroke suffer from steeper cognitive decline even before stroke onset (8, 9). That is, for some stroke patients, their cognition had already declined before the stroke. An explanation was the long-term exposure to cardiovascular risk factors before stroke (7, 10). A more severe stroke is more harmful to post-stroke cognition. Meanwhile, cardiovascular risk factors are reported to be associated with stroke and cognitive function (11). It is unclear whether the risk factors harm cognition during the long term before stroke or indirectly affect cognition by stroke severity (risk factors affect stroke severity, which in turn affects post-stroke cognition), or both.

Cardiovascular disease and stroke severity have been of great interest in studies investigating post-stroke cognitive function for the following reasons. Patients with stroke have more cardiovascular comorbidities (12). They are well-known risk factors for dementia (13, 14). Among healthy populations, hypertension and diabetes increase the risk of stroke and are associated with cross-sectionally lower cognition, cognitive decline, and incident dementia. Moreover, the heart and brain communicate intensively regarding cognition. However, among stroke survivors, the previous results are inconclusive or conflicting (15, 16). For example, previous studies found that the combination of hypertension or diabetes may be detrimental or even beneficial to cognitive function among stroke patients (17–20). The possible mechanisms include collateral circulation (21). The combined effect is still worth investigating. Furthermore, most studies analyzed the associations between risk factors and post-stroke cognition through multiple regression, but the interaction relationship and multiple routes are seldom investigated.

Here, we introduced a moderated mediation model to explore the association. The moderated mediation model is an appropriate statistical method for understanding the complex relationships between variables by calculating the mediated effect and moderated effect (11). In the mediating effect model, the independent variable is related to the mediating variable, while the mediating variable, in turn, is related to the dependent variable. In other words, it learns whether intervention in the mediating variable could change the effect of the independent variable on the dependent variable. In this study, the model considered the direct effects of risk factors (independent variable) on post-stroke cognition (dependent variable) and the indirect effects of risk factors on post-stroke cognition through changing stroke severity (mediating variable), which also affects post-stroke cognition. The moderating model hypothesizes that the moderating variable influences the direction or extent of the association between the independent variable and dependent variable. The moderating effect is assumed to occur while the direction or extent of the association between the independent and dependent variables changes according to the existence or level of the moderating variable. For example, the moderating effect of hypertension or diabetes was considered to exist while the combination of them changed the extent of the relationship between risk factors and post-stroke cognition.

Till now, only a few studies have learned the moderated and mediated effects in the domain of the post-stroke cognitive function (22–25). Drozdowska et al. (23) studied the moderated and mediated effects of cardiovascular disease on post-stroke cognitive impairment (PSCI) and found that some risk factors were indirectly associated with cognition after stroke (23). To the best of our knowledge, these associations have not been explored in China. The Chinese elders had a lower education level relative to those in Europe and the United States, resulting in less “cognitive reserve.” We hypothesized that less cognitive reserve might lead to different patterns of association (26, 27). The aim of our study was to answer the following questions: (1) Are cardiovascular risk factors directly associated with post-stroke cognitive function or (2) indirectly associated with post-stroke cognitive function through the mediating effect of stroke severity? (3) Do hypertension and diabetes moderate the relationships between cardiovascular risk factors and post-stroke cognitive function?

## Materials and methods

### Study design and participants

This was a retrospective study from a stroke neuropsychological database. The database included patients with ischemic stroke who were admitted to the stroke unit of the First Affiliated Hospital of Soochow University. Every patient in the stroke unit was provided with standardized treatment and high-dependency clinical and nursing care (28). During June 2020 and May 2021, three senior vascular neurologists (YZ, XT, and SD) collected demographic and clinical data and conducted standardized cognitive assessments for all study patients during admission. The inclusion criteria were (1) diagnosis of ischemic stroke was confirmed after admission by CT or MRI (29) and (2) patients were admitted within 7 days of illness. The exclusion criteria were (23, 30, 31) (1) patients were unable to complete the cognitive test due to existing impairment, such as aphasia. (2) Patients had disturbance of consciousness caused by a severe stroke. (3) A history of or current major depression, as determined by clinical reports or PHQ-9 of >9.

### Standard protocol approvals

All protocols followed those outlined in the Declaration of Helsinki and were approved by the Institutional Review Board of the First Affiliated Hospital of Soochow University Hospital (IRB No. 2021-172). In reporting our study, we followed Strengthening the Reporting of Observational Studies in Epidemiology (STROBE) guidelines (32).

### Data availability statement

Data were entered into the stroke registration system of the First Affiliated Hospital of Soochow University (SR-FHSU). Researchers could obtain data after the approval of the corresponding author and the ethics committee of the hospital.

### Predictors

Predictors included demographic factors and cardiovascular risk factors. Demographic factors included age, sex, education, and current smoking (13). Age was treated as a continuous variable. Education level included the following categories: primary school or less, middle school, high school, and bachelor's degree or higher. As almost all patients lived in the city where the hospital is based, we did not register living areas. Cardiovascular risk factors were associated with post-stroke dementia, hypertension, diabetes mellitus, previous stroke, vascular disease (peripheral and coronary), and atrial fibrillation (AF). The risk factors were collected by self-reported medical history and would be reevaluated during admission. After admission, due to the lack of previous medical records, we did not differentiate a previous transient ischemic attack (TIA) and a previous stroke.

### Mediators

Every patient was evaluated by the National Institute of Health Stroke Scale (NHISS) immediately upon arriving at the stroke unit. To achieve a more parsimonious model, we categorized the NHISS into four groups, namely, no stroke signs (0), minor stroke (1–4), moderate stroke (5–15), and severe stroke (16–42).

### Cognitive outcome

Cognitive function was evaluated within 7 days of ischemic stroke or TIA. It was measured by the Chinese version of the Mini-Mental State Examination (MMSE) (33). We treated MMSE scores as continuous variables.

The MMSE is a widely used tool for the assessment of cognitive function in older participants. It reflects five aspects of cognitive function, namely, orientation, registration, attention and calculation, recall, and language. The total score of MMSE ranges from 0 to 30.

### Statistical analysis

As mediation analyses assume an actual temporal order, we constructed the variables in the following orders. Stroke severity regressed on the nine predictors (including demographic and cardiovascular risk factors). Cognitive function regressed on stroke severity and regressed on the nine predictors. This reflected the direct effects of predictors and stroke severity on cognitive function and the indirect effect of predictors on cognitive function mediated by stroke severity. To avoid overfitting, we retained all predictors, regardless of whether the path was significant (34).

**Figure 1:**
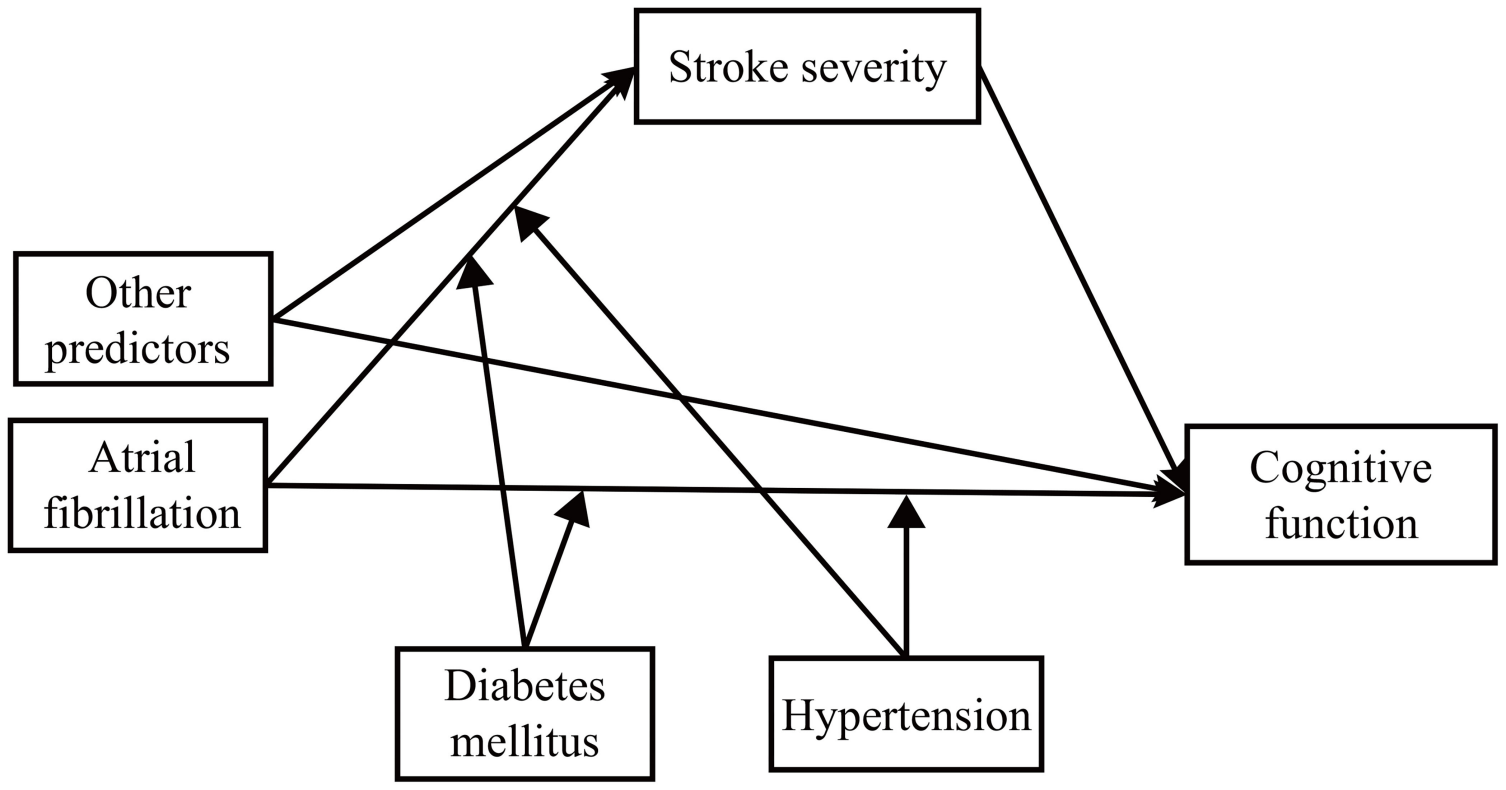
The theoretical framework model gram. We examined the moderating effect on atrial fibrillation, vascular disease, and previous stroke separately.

**Figure 2:**
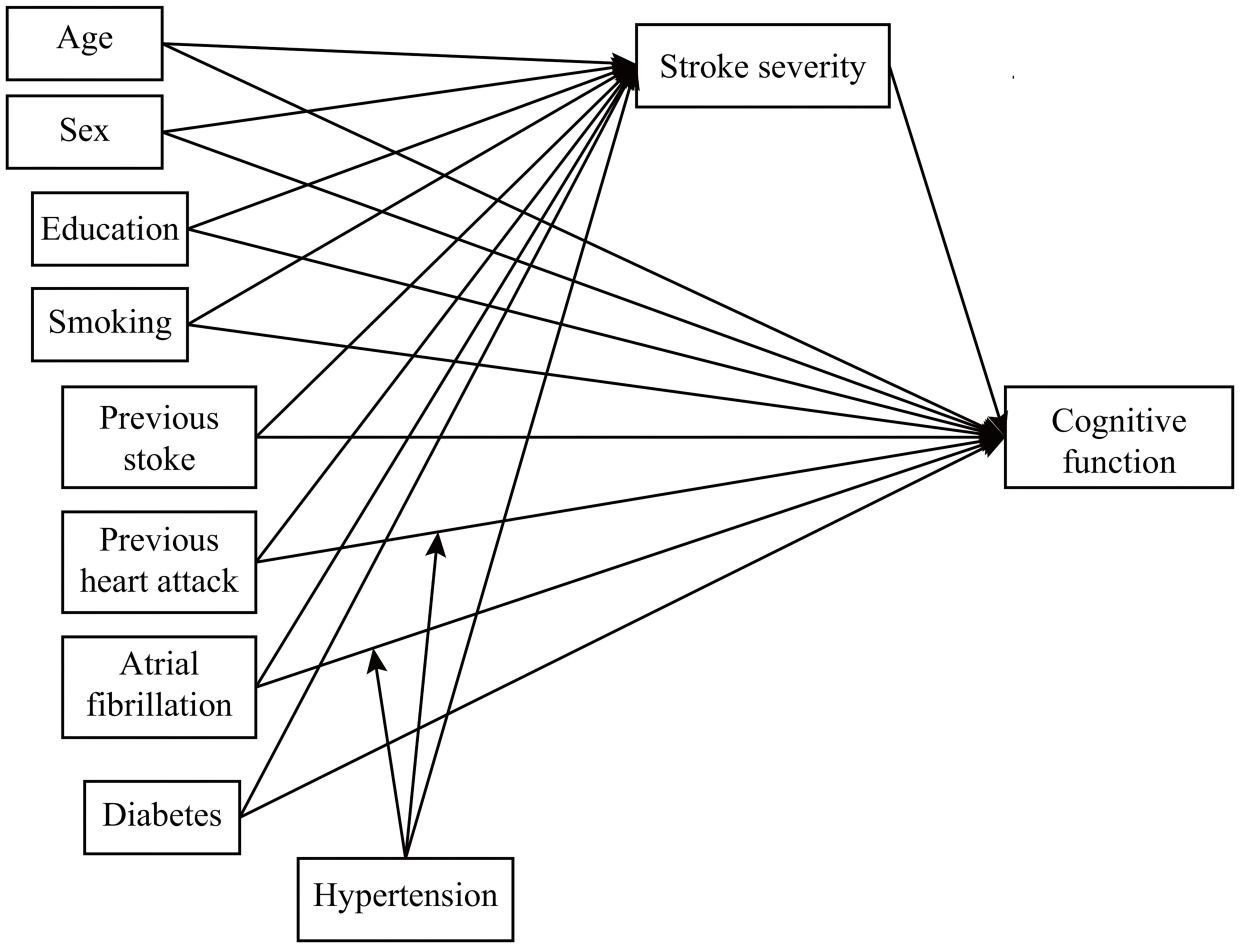
The final diagram of the moderated and mediation model.

We developed a second-stage dual moderated mediation model. First, we assumed that hypertension and diabetes might moderate the following paths: (1) the direct path between predictor and outcome and (2) the mediator path between predictor and mediator. That is, to what extent do hypertension and diabetes (1) change the direct effect of predictors on cognitive function and (2) change the indirect effect of predictors on cognitive function through stroke severity? We conducted the second-stage dual moderated mediation model for AF, vascular disease, and the previous stroke separately (Figure 1). For example, while exploring the moderated effect on AF, the model fitted four situations (presence or absence of hypertension × presence or absence of diabetes) (35). We then explored the moderating effect on the other two predictors separately. Second, we removed interaction terms with a *p*-value over 0.2 (36). In the final model (Figure 2), we kept two interaction terms (arrow pointed from hypertension to the arrow between AF and cognitive function and the arrow between vascular disease and cognitive function).

The dependent variables were all continuous; therefore, we used the maximum likelihood (ML) estimation method. It is not constricted to a normal distribution and offers better precision for calculating confidence intervals (CIs). A 1,000-replication bootstrapping process was used to estimate the CI (37, 38). Structural equation modeling was performed with Mplus 8.3 (Muthén & Muthén) (39, 40).

## Results

A total of 350 patients with ischemic stroke were included in our study. The baseline characteristics are shown in Table 1. The mean ± SD age of all participants was 63.9 ± 11.3 years; 65.1% of them were men. Half of the participants had not finished primary school. The median (interquartile range) NHISS score was 3 (1, 5). The mean±SD MMSE score of all participants was 22.6 ± 6.1.

**Table 1:** Demographic, clinical, and cognitive data of all participants (*n* = 350).

**Variable**	
Age (years)	
Median (IQR)	65 (57, 72)
Mean (SD)	63.9 (11.3)
	
Male *n* (%)	228 (65.1)
	
Education *n* (%)	
Primary school or less	177 (50.6)
Middle school	92 (26.3)
High school	52 (14.9)
Master or higher	29 (8.3)
	
Smoking *n* (%)	114 (32.5)
	
Hypertension *n* (%)	278 (79.4)
	
Diabetes mellitus *n* (%)	125 (35.7)
	
Previous stroke *n* (%)	77 (22.0)
	
Atrial fibrillation *n* (%)	37 (10.6)
	
Vascular disease *n* (%)	38 (10.7)
	
Stroke severity (NHISS score)	
Range	0–25
Median (IQR)	3 (1, 5)
Mean (SD)	3.7 (3.6)
No stroke signs	48 (13.7)
Minor stroke	195 (55.7)
Moderate stroke	104 (29.7)
Severe stroke	3 (0.9)
	
Cognitive function (MMSE score)	
Range	2–30
Median (IQR)	25.0 (19.0, 28.0)
Mean (SD)	22.6 (6.1)

### Final model contracture

First, three individual two-stage moderated mediation models were fitted to evaluate the moderation effect. Second, we opted to retain two interaction terms in the final model. We considered hypertension as a moderator for the effects of AFs and vascular disease on cognitive function. The final model is shown in Figure 2. The overall model showed excellent fitting, with SMSR = 0.002.

### Mediation effects

For direct associations, there was a statistically significant association between the mediator and cognitive function. More severe stroke was directly associated with a lower MMSE score (β = −1.994; 95% CI −2.492, −1.496; *p* < 0.001). Age, sex, education, hypertension, and AF were directly related to cognitive function (Table 2). Also, 1 year of aging was associated with a 0.122-point decrease in MMSE score (β = −0.122; 95% CI −0.149, −0.095; *p* < 0.001). Men had high MMSE scores (β = 2.502; 95% CI 1.935, 3.069; *p* < 0.001). Higher education level was associated with a high score (β = 1.341; 95% CI 1.041, 1.641; *p* < 0.001). Patients with AF were also correlated with a lower score (β = −4.092; 95% CI −5.595, −2.229; *p* = 0.028). Vascular disease was associated with higher cognitive function (β = 1.951; 95% CI 0.995, 2.907; *p* = 0.041). Smoking, hypertension, diabetes mellitus, or previous stroke was not significantly associated with cognitive function.

**Table 2:** Direct associations between predictors and cognitive function.

	**Unstandardized** ** coefficient** ** (95% CI)**	***P*-value**
Age	**–**0.122 (**–**0.149, **–**0.095)	<0.001
Sex (male)	2.502 (1.935, 3.069)	<0.001
Education	1.341 (1.041, 1.641)	<0.001
Smoking	0.664 (**–**1.198, 2.629)	0.378
Hypertension	**–**1.219 (**–**1.907, **–**0.351)	0.117
Diabetes mellitus	**–**0.037 (**–**0.948, 0.273)	0.581
Previous stroke	**–**0.591 (**–**1.337, 0.155)	0.428
Atrial fibrillation	**–**4.092 (**–**5.955, **–**2.229)	0.028
Vascular disease	1.951 (0.995, 2.907)	0.041
Atrial fibrillation × hypertension	**–**3.580 (**–**4.656, **–**2.504)	<0.001
Vascular disease × hypertension	2.464 (0.911, 4.017)	0.113

For indirect associations, higher education level (β = 0.227; 95% CI 0.135, 0.319; *p* = 0.014) and hypertension (β = 0.580; 95% CI 0.351, 0.809; *p* = 0.011) were associated with higher cognitive function through stroke severity (Table 3).

**Table 3:** Indirect associations between predictors and cognitive function.

	**Unstandardized coefficient (95% CI)**	***P*–value**
Variable		
Age	0.013 (0.006, 0.020)	0.082
Sex (male)	**–**0.049 (**–**0.192, 0.094)	0.732
Education	0.227 (0.135, 0.319)	0.014
Smoking	**–**0.144 (**–**0.744, 0.218)	0.381
Hypertension	0.580 (0.351, 0.809)	0.011
Diabetes mellitus	**–**0.055 (**–**0.203, 0.093)	0.708
Previous stroke	**–**0.103 (**–**0.286, 0.080)	0.575
Atrial fibrillation	**–**0.397 (**–**0.673, **–**0.121)	0.151
Vascular disease	0.016 (**–**0.185, 0.217)	0.936

### Moderation effects

The direct effects of AF and vascular disease could be moderated by hypertension. A combination of hypertension could decrease the magnitude of the negative association between AF and cognitive function (β changed from −4.092 to −3.580). There was also a trend that hypertension increased the magnitude of the positive association between cognitive function and vascular disease (β = 2.464; 95% CI 0.911, 4.017; *p* = 0.113). However, this association did not reach statistical significance.

## Discussion

Using a real-world sample of stroke unit patients, this article investigated the moderated and mediated associations between cardiovascular risk factors, stroke severity, and cognition. Age, female sex, AF, and stroke severity were directly associated with lower cognitive function after stroke. Vascular disease was directly associated with better cognition after stroke. For patients with AF or vascular disease, there was a trend that a combination of hypertension was associated with better cognitive function.

We achieved results different from the previous study (23). Drozdowska reported that age and AF indirectly correlated with lower cognitive function through stroke severity. In our data set, age and AF were directly associated with cognition. We further found that hypertension decreased the magnitude of the negative association between AF and post-stroke cognitive function. Generally, AF was associated with more severe stroke and thus indirectly affects cognition (41, 42). AF is strongly correlated with cardioembolic stroke. Patients who experienced cardioembolic stroke tended to be aphasia or unconsciousness. This also explains why the incidence rate of AF in our study was lower than that in other stroke data sets (~ 17%). Therefore, our results could only reflect that AF was directly associated with cognition among relatively healthier stroke survivors. Moreover, it was reasonable that other predictors were not correlated with stroke severity (23, 42).

The direct association between stroke severity and cognitive function during the acute phase after stroke was more relevant to the acute brain damage (i.e., lesions) and the subsequent immune response (i.e., the inflammatory factors) (7). A Rotterdam study included 1,443 participants with stroke (mean age at a stroke: 80.3 years), whose cognitive function had been assessed 10 years before stroke onset and several years after stroke. Compared with the control group without stroke, those with stroke had shown a faster decline 10 years before the stroke. In other words, even before the stroke, the patients with stroke had lower cognitive scores. In the acute phase after stroke, the participants with stroke exhibited cognitive decline, not surprisingly. We hypothesized that the long-term cognitive decline before stroke could explain the direct association between risk factors before the stroke. Those who were older, women, or with lower education might have lower cognitive scores before the stroke, regardless of the severity of the stroke. A similar study from the UK (participants with stroke: 694; mean follow-up, 8.2 years) thought that older patients with stroke showed more cognitive decline in the acute phase after stroke (43). Older patients are more likely to have the neurodegenerative disease (i.e., Alzheimer's disease-related pathology) and comorbidities, which may amplify the stroke injury (44, 45). Inversely, stroke could exacerbate age-related neurodegenerative pathology (46). In this article, education was directly and indirectly associated with lower cognition *via* stroke severity during the acute phase after stroke. A meta-analysis in 2009 included 79 studies learning risk factors for PSCI, 11 of which reported that lower education was a risk factor (pooled OR of the 11 studies, 2.5), while 24 of which reported that female sex was a risk factor (pooled OR of the 24 studies, 1.3) (11). A lower educational level means less cognitive reserve. Patients with less cognitive reserve already showed lower cognitive scores before the stroke. In the acute phase after stroke, education also seemed to modify the effect of stroke on cognitive decline (47). Patients who received better education suffered less from cardiovascular disease due to various reasons, including a healthy lifestyle, safe working conditions, and better access to healthcare before stroke (48). The negative associations between sex and post-stroke cognitive function were in accordance with results from other studies in China. A national representative epidemiological study, using data from the China Health and Retirement Longitudinal Study, attributed the cognitive difference to schooling, family, and community levels of economic resources (49). Therefore, female patients had lower cognition than male patients before the stroke, which could explain the direct associations. After stroke onset, female stroke survivors were reported to experience faster cognitive decline after stroke than male stroke survivors. The mechanism underlying the gender difference after stroke remains to be elucidated. Sex differences in the expression of brain-derived neurotrophic factors, sex hormones, and stroke subtype were involved in the gender difference in cognition after stroke (50).

Atrial fibrillation was independently associated with lower cognitive function among participants with or without stroke (51–53). It results in a series of mechanisms that would cause lower cognition, such as cerebral hypoperfusion, inflammatory responses, silent ischemia, reduced brain volumes, and cerebral microbleeds. The effect of blood pressure on clinical outcomes was contradictory. Higher blood pressure might increase infarct volume, brain edema, and hemorrhagic transformation (54). A study including 306 patients with stroke learned the association between baseline blood pressure and outcomes. Among a subgroup, higher blood pressure was associated with improved collateral flow, decreased infarct growth, and better functional outcomes (55). This was a potential explanation of our findings. For patients with AF or vascular disease, a combination of hypertension might bring better collateral flow and higher cognitive scores. Future studies are needed to explore objective evidence. The association between vascular disease and cognitive function is still controversial (56, 57).

To the best of our knowledge, this was the first study to identify the moderated and mediated association between cardiovascular risk factors, stroke severity, and cognitive function in Chinese patients with stroke. We highlighted the importance of comorbidities while learning cognitive function after stroke. However, we also had several disadvantages. First, we only performed cognitive tests within 7 days after stroke. Future studies could examine the effects of risk factors on cognitive function 3–6 months after stroke. Second, our data set included a few patients with severe stroke. However, patients suffering from severe stroke often have problems with consciousness or aphasia. The disability prevented us from performing cognitive tests. Hence, our results could only reflect situations in relatively healthy stroke survivors. Third, we could not distinguish previous TIA from the previous stroke. We were not able to obtain the previous medical records of our patients who were not admitted to our hospital before. Furthermore, in our district, patients had a relatively lower education level and thus had a lower awareness rate of the previous history. Fourth, our study participants were from China. The generalizability of the results to other countries might be a concern. Fifth, due to a lack of medical records, we could not identify those with mild cognitive impairment or dementia before the stroke. Future studies could obtain information on pre-stroke cognition through questionnaires like the Informant Questionnaire of Cognitive Decline in the Elderly.

## Conclusion

Our research explored the complex relationships between cardiovascular risk factors, stroke severity, and cognitive function. Age and AF were directly associated with lower cognition after stroke. Hypertension could decrease the magnitude of the negative association between AF and post-stroke cognition. Future studies are needed to learn whether the associations are causal.

## Data availability statement

Data were entered into the stroke registration system of the First Affiliated Hospital of Soochow University (SR-FHSU). Researchers could obtain data after the approval of the corresponding author and the ethics committee of the hospital.

## Ethics statement

All protocols followed those outlined in the Declaration of Helsinki and were approved by the Institutional Review Board of The First Affiliated Hospital of Soochow University Hospital (IRB No. 2021-172). In reporting our study, we followed Strengthening the Reporting of Observational Studies in Epidemiology (STROBE) guidelines. Written informed consent for participation was not required for this study in accordance with the national legislation and the institutional requirements.

## Author contributions

QF and JH contributed to the conception and design of the study. SD, YZ, and XT performed cognitive tests and collected medical data. JH performed the statistical analysis. JH and LC wrote the first draft of the manuscript. QF and XT reviewed the manuscript. All authors approved the final version of the paper.

## Funding

This study was supported by grants from the National Science Foundation of China (82071300), Health Expert Training Program of Suzhou-Gusu District (GSWS2020002), and Medical Team Introduction Program of Soochow (SZYJTD201802). It was supported by the National Natural Science Foundation of China (no. 82001125, XT) and Natural Science Foundation of Jiangsu Province (no. BK20180201 XT).

## Conflict of interest

The authors declare that the research was conducted in the absence of any commercial or financial relationships that could be construed as a potential conflict of interest.

## Publisher's note

All claims expressed in this article are solely those of the authors and do not necessarily represent those of their affiliated organizations, or those of the publisher, the editors and the reviewers. Any product that may be evaluated in this article, or claim that may be made by its manufacturer, is not guaranteed or endorsed by the publisher.
