# Supplementary material for: How do cardiovascular risk factors correlate with post-stroke cognitive function: Directly or indirectly through stroke severity?
Source: Front Neurol. 2022 Aug 5;13:917295. doi: 10.3389/fneur.2022.917295 (PMC9389173; doi:10.3389/fneur.2022.917295)
Supplement: Supplementary file 1 [file Table_1.DOCX]

Supplementary Material

Table S1 Direct associations between predictors and mediators.

|  | Unstandardized coefficient (95% CI) | P value |
| --- | --- | --- |
| Age | -0.006 (-0.009, -0.003) | 0.052 |
| Sex (male) | 0.025 (-0.048, 0.098) | 0.737 |
| Education | -0.114 (-0.150, -0.078) | 0.002 |
| Smoking | 0.093 (-0.147, 0.340) | 0.314 |
| Hypertension | -0.291 (-0.382, 0.020) | 0.001 |
| Diabetes mellitus | 0.028 (-0.043, 0.099) | 0.697 |
| Previous stroke | 0.052 (-0.040, 0.144) | 0.574 |
| Atrial fibrillation | 0.199 (0.071, 0.327) | 0.119 |
| Vascular disease | -0.008 (-0.107, 0.091) | 0.935 |
